# In Vitro Activity of the Bacteriophage Endolysin HY-133 against *Staphylococcus aureus* Small-Colony Variants and Their Corresponding Wild Types

**DOI:** 10.3390/ijms20030716

**Published:** 2019-02-07

**Authors:** Nina Schleimer, Ursula Kaspar, Dennis Knaack, Christof von Eiff, Sonja Molinaro, Holger Grallert, Evgeny A. Idelevich, Karsten Becker

**Affiliations:** 1 Institute of Medical Microbiology, University Hospital Münster (UKM), 48149 Münster, Germany; nina.schleimer@ukmuenster.de (N.S.); ursula-kaspar@ukmuenster.de (U.K.); dennis.knaack@sfh-muenster.de (D.K.); christof.voneiff@pfizer.com (C.v.E.); evgeny.idelevich@ukmuenster.de (E.A.I.); 2 HYpharm GmbH, 82347 Bernried, Germany; s.molinaro@microcoat.de (S.M.); holger.grallert@hyglos.de (H.G.)

**Keywords:** *Staphylococcus aureus*, small-colony variants, oxacillin, endolysin, HY-133, susceptibility, nasal decolonization, functional resistance

## Abstract

Nasal carriage of methicillin-susceptible (MSSA) and methicillin-resistant *Staphylococcus aureus* (MRSA) represents both a source and a risk factor for subsequent infections. However, existing MRSA decolonization strategies and antibiotic treatment options are hampered by the duration of administration and particularly by the emergence of resistance. Moreover, beyond classical resistance mechanisms, functional resistance as the formation of the small-colony variant (SCV) phenotype may also impair the course and treatment of *S*. *aureus* infections. For the recombinant bacteriophage endolysin HY-133, rapid bactericidal and highly selective in vitro activities against MSSA and MRSA has been shown. In order to assess the in vitro efficacy of HY-133 against the SCV phenotype, minimal inhibitory (MIC) and minimal bactericidal concentrations (MBC) were evaluated on clinical SCVs, their isogenic wild types, as well as on genetically derived and gentamicin-selected SCVs. For all strains and growth phases, HY-133 MIC and MBC ranged between 0.12 and 1 mg/L. Time-kill studies revealed a fast-acting bactericidal activity of HY-133 resulting in a ≥3 − log_10_ decrease in CFU/mL within 1 h compared to oxacillin, which required 4–24 h. Since the mode of action of HY-133 was independent of growth phase, resistance pattern, and phenotype, it is a promising candidate for future *S. aureus* decolonization strategies comprising rapid activity against phenotypic variants exhibiting functional resistance.

## 1. Introduction

*Staphylococcus aureus* represents a widely disseminated and invasive pathogen causing clinically important and life-threatening infectious diseases [1,2]. Since *S. aureus* colonizes 30% or more of all humans, primarily the vestibules of the nasal cavity, the risk for subsequent infections originating from the patients’ own flora is significantly increased [2,3,4,5,6,7]. The possession of healthcare- (HA-), community- (CA-), and livestock-associated (LA-) methicillin-resistant *S. aureus* (MRSA) drastically aggravates the treatment by limiting the therapeutic options and necessitates expansive preventive measures, therefore resulting in additional morbidity, mortality, and costs [1,8,9]. Resistances also emerged against remaining or newly developed antibiotic classes with anti-MRSA activity such as glycopeptides, oxazolidinones, cyclic lipopeptides, and anti-MRSA cephalosporins, often shortly after their introduction [10,11,12,13]. Current strategies to prevent nosocomial *S. aureus* infections, particularly those due to MRSA, are frequently based on the topical application of the antibiotic agent mupirocin [14,15,16], but a considerable number of patients show recolonization [17,18,19]. Possible reasons include its bacteriostatic mode of action and the rising emergence of resistance against this substance [20,21,22,23,24].

Beyond these classical resistance mechanisms, resistance based on microbial biofilm lifestyle or the formation of the small-colony variant (SCV) phenotype may render microbial isolates functionally resistant even if they are susceptible to antibiotics. Moreover, the administration of antibiotics, in particular long-term exposure, may select for the generation of SCVs [25,26]. The naturally occurring SCV phenotype is capable of evading the host´s immune response by switching from an extracellular to an intracellular lifestyle, resulting in chronic, relapsing, and often therapy-refractory infections [27,28,29]. Furthermore, nasal colonization by SCVs and intracellular persistence in nasal epithelium have been shown [30,31].

The ability of SCVs to persist within eukaryotic cells and their changed physiology is also of particular therapeutic significance, as the activity of many antibiotic compounds may be attenuated. In particular, β-lactams, which in general represent the most effective anti-staphylococcal agents, are not bioavailable in the intracellular milieu [32]. Moreover, SCVs may be tolerant against β-lactams (e.g., oxacillin). The tolerance phenomenon was already shown for dormant and slow-growing cells and might also result in treatment failure [33,34,35,36,37]. Reduced susceptibilities of staphylococcal SCVs to antifolate antibiotics, aminoglycosides, and cationic antimicrobial compounds are also common traits of this phenotype compared to isolates displaying the normal phenotype [38,39]. Compounds of other antibiotic classes usually show minimal inhibitory concentrations that are similar to those measured for strains with a normal phenotype, but they are frequently less bactericidal in the case of SCVs [40,41]. Typical SCV colonies differ from the parental wild type (WT) not only in slower growth with less or no pigmentation and hemolytic activity, but also in several biochemical traits, such as altered expression of virulence factors, decreased respiration, as well as different forms of auxotrophisms, e.g., for hemin, menadione, and/or thymidine [42,43,44]. Moreover, SCVs are characterized by major ultrastructural alterations of the cell wall and cell separation structures [45,46,47].

Obviously, the limited treatment options of *S. aureus* infections due to classical and functional resistance mechanisms necessitate alternative prophylactic and therapeutic options. Bacteriophage-derived endolysins exhibit the ability to disrupt bacterial cell walls in a highly specific manner [48]. Recombinant endolysins are characterized by a modular design comprising an enzymatically active domain (EAD) connected by a short linker region with specific cell wall recognition site (cell wall-binding domain, CBD) [49]. In the pre-antibiotic era, phages were successfully used for the treatment of various bacterial infections; however, the introduction of antibiotics stopped their use in Western medicine [50]. Only in recent years has research on this subject been revived, resulting in numerous recombinant endolysins that are active in vitro and/or in vivo against several gram-negative and gram-positive bacteria including *Pseudomonas aeruginosa* [51], *Streptococcus* spp. [52,53,54,55], and *Staphylococcus* spp. [56,57,58,59,60,61,62]. Moreover, phage endolysins were shown to be active against difficult-to-treat bacterial infections caused by phenotypically resistant bacterial populations, e.g., by being embedded in biofilms [63,64,65]. 

The recombinant endolysin HY-133 (HYpharm GmbH, Bernried, Germany) used in this study represents a stability-optimized derivative of endolysin PRF-119 [59,60]. The modular structure of HY-133 comprises a CBD from lysostaphin, responsible for the specific attachment to the peptidoglycan of *S. aureus* [59,62]. Its EAD consists of the cysteine, histidine-dependent amidohydrolase/peptidase (CHAP) originating from the recombinant endolysin LysK of phage K [62,66]. Within the peptidoglycan of *S. aureus*, this CHAP domain cleaves between d-alanine at the termini of the tetrapeptide and glycine of the pentaglycine crossbridge, and is thereby responsible for the lytic mode of action [67]. HY-133 was previously shown to be highly active against methicillin-susceptible *Staphylococcus aureus* (MSSA) and MRSA isolates [59,60,61,62]. However, the efficacy of Hy-133 against SCVs has yet to be determined. Here, the activity of HY-133 by the determination of the minimal inhibitory (MIC) and minimal bactericidal concentrations (MBC) and time-kill analyses was systematically analyzed against a well-characterized collection of clinically derived MSSA SCVs and mutants displaying the SCV phenotype in comparison with their parental WTs. Oxacillin was used as comparator agent.

## 2. Results

### 2.1. Genotyping by Pulsed-Field Gel Electrophoresis (PFGE)

We previously demonstrated the very fast and high bactericidal in vitro activity of the recombinant endolysin HY-133 against several clinical MSSA and (LA-)MRSA isolates [59,60,61,62]. To further investigate the activity of HY-133, it was challenged with 12 clinical *S. aureus* strain pairs, each comprising WT and stable SCV phenotypes. Since the pulsed-field gel electrophoresis (PFGE) fragment patterns of the WT-SCV pairs were indistinguishable or varied in only one band, all strain pairs were considered clonally identical according to published guidelines [68] (data not shown).

### 2.2. Antimicrobial Susceptibility Testing

MIC_50_, MBC_50_, as well as MIC_90_ and MBC_90_ with the corresponding ranges were calculated for the different growth phases and phenotypes for HY-133 and oxacillin and are listed in Table 1 for the 12 clinical strain pairs (*n* = 24). Table 2 lists the MIC and MBC values evaluated for the isogenic 6850 triplet consisting of the WT isolate 6850, its gentamicin-selected SCV JB1, and its *hemB* mutant SCV IIb13. MIC_50/90_ and MBC_50/90_ for HY-133 tested against the 12 clinical strain pairs were similar for the different phenotypes or growth phases. The same applied for oxacillin which, however, exhibited slightly higher MIC_50/90_ and MBC_50/90_ values for clinical strains than HY-133 (Table 1).

Within the 6850 triplet, the MIC and MBC of HY-133 were similar for the different phenotypes (0.12–0.25 mg/L) when measured from cultures in stationary growth phase. Measurements from logarithmic growth cultures revealed the same MIC and MBC values against 6850 (WT) and IIb13 (SCV), being 0.25 mg/L for both strains and reflecting values detected during the stationary phase. However, HY-133 MIC and MBC values measured against the gentamicin-selected SCV JB1 were increased (1 mg/L) in this growth phase. For oxacillin, MIC and MBC were substantially lower against the 6850-derived SCVs JB1 and IIb13 than against the corresponding WT and the clinical strains, irrespective of the growth phase (Table 2).

Furthermore, for HY-133, the median MBCs were higher than the median MICs in 4.2% (*n* = 2/48) of cases with an MBC:MIC ratio of 2. In either case, the SCV phenotype tested from stationary growth cultures was affected. However, when strains were treated with oxacillin, 20.8% (*n* = 10/48) showed a higher median MBC than MIC with 40% (*n* = 4/10) being WTs and 60% (*n* = 6/10) being SCVs, respectively, and an MBC:MIC ratio of 2 in each case.

The quality control (QC) strain ATCC 29213 showed oxacillin values in the range as required by the Clinical & Laboratory Standards Institute (CLSI) guidelines [69]. There is no official QC range available for HY-133, but the MICs detected for the QC strain ATCC 29213 were always within the in-house established limits.

### 2.3. Time-Kill Studies

Time-kill curves were performed for the two representative clinical strain pairs OM299 and 4652 as well as for the strains of triplet 6850. Time-kill curves revealed distinct growth characteristics of the two phenotypes with clinical and 6850-derived SCVs exhibiting a clear growth retardation compared to the WTs, as described in previous studies [42,43,70]. For the clinical strain pairs OM299 and 4652, this difference in growth was more pronounced for strain pair OM299. 

**Figure 1:**
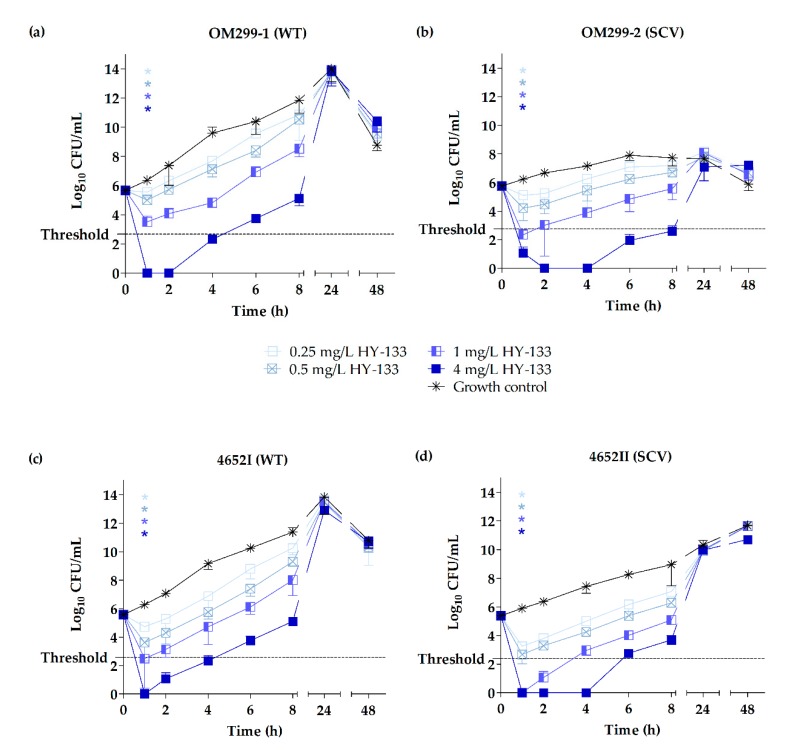
Time-kill curves of HY-133 shown by plots of mean values for the log_10_ of the numbers of CFU/mL versus time for two representative clinical *Staphylococcus aureus* WTs (**a**,**c**) and corresponding SCVs (**b**,**d**) tested against HY-133. The threshold implicates a ≥3 − log_10_ decrease in CFU/mL. Time-kill curves for each strain were performed in triplicate (mean ± standard deviation). Asterisks denote statistical difference of the respective concentration of HY-133 used (defined by matching colors) with respect to the untreated growth control at 1 h; *p* ≤ 0.001 by one-way ANOVA.

**Figure 2:**
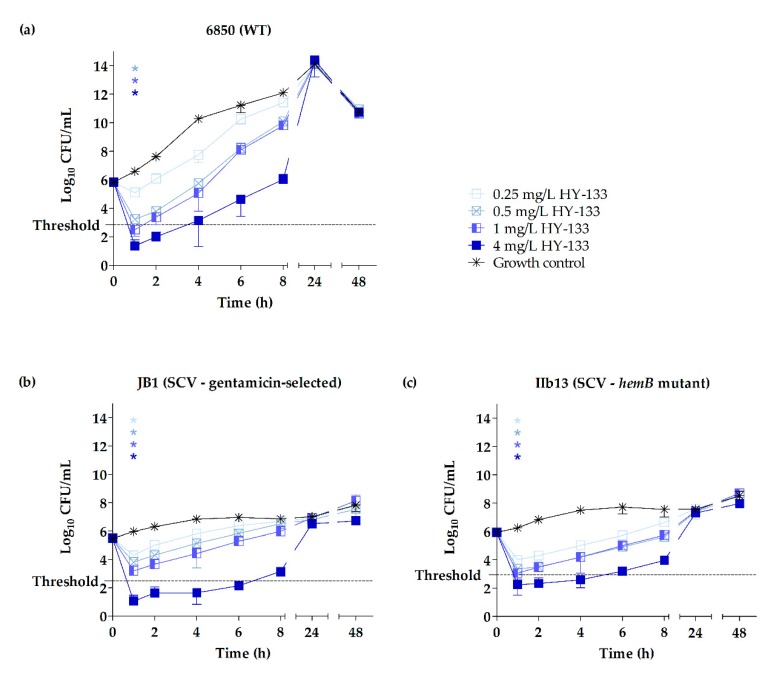
Time-kill curves of HY-133 shown by plots of mean values for the log_10_ of the numbers of CFU/mL versus time for the highly cytotoxic and clinically virulent *S. aureus* WT strain 6850 (**a**) and corresponding SCVs JB1 (**b**) and IIb13 (**c**) tested against HY-133. The threshold implicates a ≥3 − log_10_ decrease in CFU/mL. Time-kill curves for each strain were performed in triplicate (mean ± standard deviation). Asterisks denote statistical difference of the respective concentration of HY-133 used (defined by matching colors) with respect to the untreated growth control at 1 h; *p* ≤ 0.001 by one-way ANOVA.

**Figure 3:**
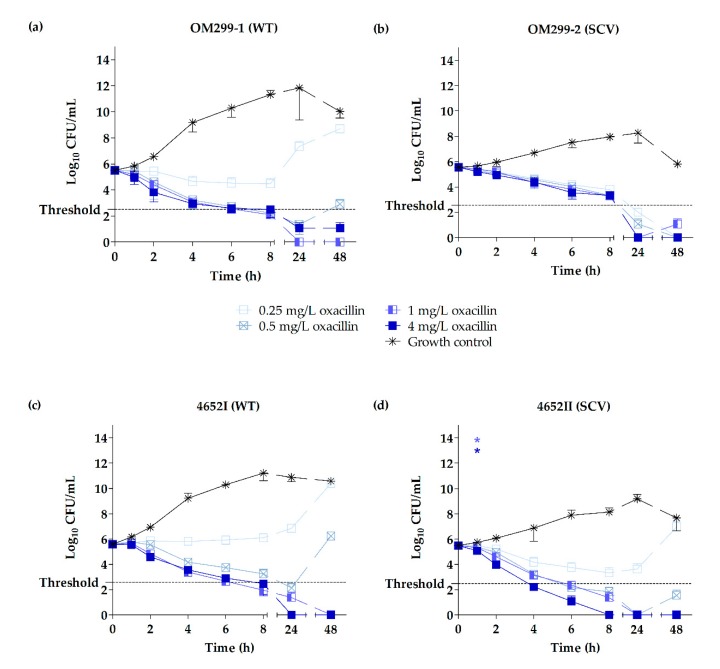
Time-kill curves of oxacillin shown by plots of mean values for the log_10_ of the numbers of CFU/mL versus time for two representative clinical *S. aureus* WTs (**a**,**c**) and corresponding SCVs (**b**,**d**) tested against oxacillin. The threshold implicates a ≥3 − log_10_ decrease in CFU/mL. Time-kill curves for each strain were performed in triplicate (mean ± standard deviation). Asterisks denote statistical difference of the respective concentration of oxacillin used (defined by matching colors) with respect to the untreated growth control at 1 h; *p* ≤ 0.001 by one-way ANOVA.

Killing kinetics of HY-133 against the clinical and 6850-derived strains revealed significant differences in the killing rates of HY-133 compared to the untreated growth controls (*p* ≤ 0.001 for all concentrations, except for 0.25 mg/L HY-133 against strain 6850), already after 1 h of incubation. At this time point, a ≥3 − log_10_ decrease in CFU/mL (99.9% killing) was shown for all strains irrespective of the phenotype when applying a concentration of 4 mg/L (Figure 1 and Figure 2, Table 3, and Table S1 in the Supplementary Material). Furthermore, this concentration led to a reduction of cell numbers below the detection limit for all clinical isolates tested for at least 1 h (Figure 1). However, regarding the strain 6850-derived triplet, neither the WT nor the in vitro-generated SCVs reached this limit (Figure 2). Corresponding levels of oxacillin (4 mg/L) were bactericidal against the clinical WTs after a time interval of 8 h. By contrast, the time until a bactericidal effect was detected in the clinical SCVs varied between 4 h for SCV 4652II and 24 h for SCV OM299-2. The detection limit for oxacillin (4 mg/L) was reached between 8–24 h for all strains and phenotypes (Figure 3, Table 3 and Table S1). After 1 h of incubation, significant differences in the killing rate of oxacillin versus the untreated growth control were observed only for the SCV strain 4652II applying 1 and 4 mg/L of the substance (*p* ≤ 0.001). A concentration of 1 mg/L of HY-133 was bactericidal for most of the strains within 1 h except for the clinical WT strain OM299-1 as well as for SCVs JB1 and IIb13 (Table 3). All other HY-133 concentrations applied caused no bactericidal effect. For oxacillin, a bactericidal effect was also reached for concentrations of 1 and 0.5 mg/L. For these concentrations, time intervals until reaching bactericidal effect differed between 6 and 24 h irrespective of the phenotype. Moreover, for SCV OM299-2, a concentration of 0.25 mg/L oxacillin was bactericidal after 24 h (Table 3).

The killing effect for HY-133 was similar for SCVs and their respective WTs (Figure 1 and Figure 2). After prolonged incubation (4–24 h for 4 mg/L HY-133), time-kill curves for HY-133 revealed a regrowth phenomenon for all tested strains and phenotypes, even for concentrations initially resulting in values below the detection limit. For clinical SCVs as well as for SCVs JB1 and IIb13, this regrowth appeared slower than for WTs when applying 4 mg/L HY-133 (Figure 1 and Figure 2). For oxacillin, regrowth above the threshold after prolonged incubation (>24 h for 0.5 mg/L oxacillin) could also be detected for concentrations less than or equal to 0.5 mg/L for both WTs, whereas for the SCVs, regrowth above the threshold could only be observed for the lowest concentration (0.25 mg/L) for strain 4652 (Figure 3).

## 3. Discussion

In recent years, several in vitro and in vivo studies revealed the high potential of endolysins for the treatment of several bacterial infections [49]. HY-133 represents one of its promissing candidates in anti-*S. aureus* treatment and prevention regimens. This recombinant phage endolysin and its precursor PRF-119 were previously shown to be highly active against different strains of MSSA, MRSA (including strains possessing *mecA*, *mecB*, or *mecC* genes), borderline oxacillin-resistant isolates, as well as isolates that were found to be resistant to ceftaroline/ceftobiprole [59,60,61,62]. Recently, we demonstrated that HY-133 is as effective against *S. aureus* isolates as mupirocin or daptomycin, with an even faster mode of killing [61].

In contrast to the vast majority of *S. aureus* clonal lineages possessing polyribitol phosphate wall teichoic acid (WTA) [71], the unusual polyglycerol phosphate WTA-producing *S. aureus* PS 187, which belongs to a distant *S. aureus* lineage (phage type 187, biotype E) often isolated from dogs [72], was less susceptible to PRF-119. Thus, changes of the cell wall composition or structure may influence the endolysin activity [59]. Also SCVs, functionally resistant to several antibiotic classes, are notorious for gross ultrastructural changes of the cell wall. As shown by transmission and scanning electron microscopy, they are characterized by the formation of pleomorphic cocci up to eight times larger than those of WTs and impaired cell separation with multiple cross walls [45,46,47,73]. Furthermore, SCVs exhibit slower growth characteristics in contrast to their parental WT isolates. Lastly, SCVs are characterized by altered metabolism [42,43,74]. As the mechanism in which HY-133 binds to the cell wall is very specific and its mode of action does not depend on the target’s metabolic processes, the analysis of its efficacy against the SCV phenotype in comparison to the corresponding WT is a further necessary step in the determination of the anti-staphylococcal activity of this substance. 

To consider the divergent growth characteristics of SCVs [27], the determination of MIC and MBC values was not only performed from cultures in stationary growth, but also from the logarithmic growth phase. MIC and MBC determinations revealed a highly bactericidal effect of HY-133 against SCVs, independent of growth phase and to the same extent as that observed for the corresponding WTs. In contrast, the endolysin encoded by LM12, a *Kayvirus* bacteriophage with broad *S. aureus* host range, was shown to exhibit growth phase-dependent activity [75]. The killing kinetics of HY-133 revealed a very fast mode of action until 99.9% of the bacterial population was killed. This rapid mode of action within 1 h was also observed for the gentamicin-selected and the genetically defined SCVs of the highly cytotoxic strain 6850. This finding reflects the observation of another study, where the exceptionally fast onset of action of HY-133 within the first minutes of application for MSSA and MRSA WT phenotypes was demonstrated [61]. The observed prolongation of the bactericidal effect for the SCVs treated with HY-133 (4 mg/L) is most likely due to the slower growth of the SCVs.

Whereas MIC and MBC values for oxacillin did not show significant differences to MIC and MBC values of HY-133, time-kill studies revealed that the onset of bactericidal effect of oxacillin (4 mg/L) was not as fast as that detected for HY-133 within 1 h and was reached only after 4 to 24 h. For strain pair OM299, including an SCV exhibiting a considerable growth retardation compared to its WT, the detected killing effect of the SCV by oxacillin was clearly delayed compared to its WT. For β-‍lactams, tolerance is a known phenomenon in slow-growing or dormant bacteria [33,34,35], which appears due to the slower assembly of the bacterial cell wall and necessitates a longer minimum duration of antimicrobial treatment to reach the same bactericidal effect [37]. However, the tolerance phenomenon can result in treatment failures [36,37]. Since the CLSI standard definition of antimicrobial tolerance is defined as an MBC:MIC ratio of ≥32 (M26-A) [76], a tolerance phenomenon could not be confirmed by the MBC:MIC ratio calculation in this case. However, according to the terminology used by Brauner et al. [37], SCV OM299-2 exhibited a longer minimum duration of treatment and therefore might be tolerant. On the contrary, SCV 4652II—exhibiting only a minor growth retardation compared to its WT—showed no signs for tolerance, suggesting that tolerance towards β-lactams might not be a general phenomenon for SCVs. Moreover, for a methicillin-resistant *menD* SCV mutant, hyper-susceptibility to β-lactams in a macrophage cell model has been shown [77]. For HY-133, no tolerance phenomenon was detected for any of the SCVs including the gentamicin-selected and genetically defined SCVs.

This study highlights one of the most relevant advantages of HY-133, i.e., its particular mode of action. The modular design of HY-133 is responsible for the species-specific cleavage of the peptidoglycan of *S. aureus*, thus inducing cellular lysis. This form of cell death occurs independently of cellular processes. In contrast, most conventional antibiotics operate by inhibiting essential cellular functions [78], e.g., inhibition of the cell wall biosynthesis by the β-lactam oxacillin. However, in SCVs, these processes can be altered, leading to failures in antibiotic treatment. Long-term treatment with trimethoprim-sulfamethoxazole (TMP-SXT) that inhibits the dihydrofolate reductase within the bacterial folate pathway may result in SCVs auxotrophic for thymidine and resistant to TMP-SXT [38]. Furthermore, SCVs with deficiencies in electron-transport show resistance to aminoglycosides. Due to their lack in membrane potential that is normally generated by electron-transport, the bacterial uptake of aminoglycosides is impaired [25,39,40,79].

For HY-133, the killing kinetics revealed an in vitro regrowth phenomenon for all strains tested appearing after prolonged incubation. This regrowth occurred even after an initial killing of 99.9% of the bacerial population. Its extent depended on both the concentration of HY-133 and the strain used. A similar in vitro regrowth phenomenon was previously shown for clinical MSSA and MRSA isolates [61]. Further investigations are warranted to understand whether the inactivation of HY-133 is an exclusive in vitro phenomenon caused, e.g., by environmental conditions (medium, shearing forces etc.) which may irreversibly degrade the endolysin, complete the consumption of HY-133, or escape by remnant susceptible cells via hiding mechanisms.

The present study demonstrates that HY-133 caused very rapid cell death independent of the rate of bacterial growth, the consistency of the cell wall, and the distinct growth phase. Although mutants resistant to the bacteriocin lysostaphin have been created [80,81], selection pressure for resistances against endolysins seems to be absent or very low [49,55,82,83]. This is most likely due to the fact that the respective regions of the bacterial cell wall are highly conserved [84,85]. Thus, since there were also no adverse side effects reported until now [86], the recombinant phage endolysin HY-133 may serve as a future narrow-spectrum antimicrobial drug used in treatment and infection prevention purposes for the eradication of normal-growing *S. aureus* cells as well as for bacterial cells with impaired growth. *S. aureus* SCVs possess the capacity to enter and persist in host cells [73,79,87,88]. Therefore, further cell-culture experiments are necessary to elucidate the cell permeability of HY-133. Recently, the penetration of mammalian cells was demonstrated for the anti-streptococcal endolysin PlyC that was able to readily kill intracellular *Streptococcus pyogenes* without damaging the host cell [86].

In summary, HY-133 was shown to be highly bactericidal in the logarithmic as well as in the stationary growth phases of all tested clinical *S. aureus* WT and SCV isogenic strain combinations, irrespective of their exhibited phenotype or genetic background. Due to its highly bactericidal activity and its high specificity, HY-133 is a promising candidate for future anti-staphylococcal therapy, decolonization, and prevention, particularly for difficult-to-treat phenotypes such as SCVs.

## 4. Materials and Methods 

### 4.1. Bacterial Strains and Maintenance

Twelve clinical *S. aureus* WT isolates and their corresponding stable SCVs were obtained from patients of the University Hospital Münster (UKM), Münster, Germany (see Table 4) [39,89,90]. Each strain pair was isolated from one patient either consecutively or concurrently. All isolates were categorized as methicillin-susceptible by (i) disk diffusion methodology (oxacillin susceptibility disks, Oxoid, Hampshire, United Kingdom; Mueller Hinton II agar, MHA, Becton Dickinson, Franklin Lakes, NJ, USA) and (ii) absence of methicillin resistance-encoding *mecA*, *mecB*, and *mecC* genes, as described elsewhere [91,92,93]. Isolates were characterized as SCVs according to the following criteria: pinpoint colonies on Columbia blood agar (BBL^TM^ Columbia agar with 5% sheep blood, Becton Dickinson, Franklin Lakes, NJ, USA) after approximately 48 h of incubation, decreased pigmentation, and reduced hemolytic activity [27]. The phenotypic stability of the SCVs was verified by parallel cultivation on Columbia blood agar throughout all experiments. Unstable SCVs showing revertants after several passages on Columbia blood agar were excluded from analysis.

Furthermore, the highly cytotoxic MSSA 6850—originally isolated from a patient with a complicated *S. aureus* skin abscess [94]—and the corresponding SCVs JB1 and IIb13 were tested (see Table 4).

Strains were cultivated on Columbia blood agar at 37 °C for 24–48 h. Unless otherwise stated, liquid cultures were grown aerobically overnight in 10 mL cation-adjusted Mueller Hinton II broth (CAMHB, Becton Dickinson, Franklin Lakes, NJ, USA) in 100-mL glass baffled flasks at 37 °C and 160 rpm.

### 4.2. Genotyping by PFGE

Insofar as this has not already been done in previous studies [39,89,90], the clonal relationship between the strain pairs was identified by *Sma*I macrorestriction analyses of total bacterial DNA and resolving the digests with the use of PFGE, as previously described [96]. Strains were considered clonal according to published guidelines [68].

### 4.3. Antimicrobial Susceptibility Testing

The MIC and MBC values of HY-133 (HYpharm GmbH, Bernried, Germany) and oxacillin (Sigma-Aldrich, St. Louis, MO, USA) were determined in sterile 96-well microplates (Greiner Bio One International, Kremsmünster, Austria) using standard CLSI broth microdilution methodology for staphylococci [69,97]. CAMHB was used for HY-133 and CAMHB with 2% NaCl for oxacillin. Both antimicrobials were used in a 2-fold dilution series with concentrations ranging from 0.016 to 8 mg/L. Direct colony suspensions from stationary phase were prepared in accordance with CLSI methodology for staphylococci [69,97]. Briefly, colonies from overnight Columbia blood agar cultures were adjusted to McFarland 0.5 in 2 mL of NaCl. Subsequently, cultures were diluted in either CAMHB for HY-133 or CAMHB with 2% NaCl for oxacillin followed by the addition of the appropriate amount of antimicrobial substance to obtain the final starting inoculum of 5 × 10^5^ CFU/mL. Furthermore, to test the activity of HY-133 and oxacillin under logarithmic growth conditions, colonies from overnight Columbia blood agar cultures were used to prepare 10 mL tryptic soy broth (TSB, Becton Dickinson, Franklin Lakes, NJ, USA) cultures with a starting OD_578_ of 0.05. After 3 h of incubation at 37 °C and 160 rpm, starting inocula of 5 × 10^5^ CFU/mL were prepared. Microplates were incubated at 37 °C for 18–20 h for HY-133 and 24 h for oxacillin.

For the evaluation of the MBC, 10 µL of culture medium from each microwell displaying the MIC and at least one concentration above were inoculated onto tryptic soy agar (TSA, Becton Dickinson, Franklin Lakes, NJ, USA) plates. After incubation at 37 °C overnight, CFUs were counted. The MBC was defined as the concentration of antimicrobial substance generating <500 CFU/mL (killing rate of 99.9%; this corresponds to a ≥3-log10 decrease in CFU/mL) [76]. MICs and MBCs were determined in triplicate and the median MIC and MBC values were calculated for further analyses. *S. aureus* ATCC 29213 was used as a QC strain. 

### 4.4. Time-Kill Studies

In addition to MIC and MBC determination, the killing kinetics of HY-133 and oxacillin were evaluated by means of time-kill curves for two representative strain pairs (OM299 and 4652). Moreover, the killing kinetics of HY-133 were determined for triplet 6850. Time-kill curves were performed in triplicate with the macrodilution method in accordance with CLSI guideline M26-A [76]. Briefly, overnight cultures were grown on Columbia blood agar plates at 37 °C. For the detection of bactericidal activity, colonies were inoculated in 10 mL TSB and incubated for 3 h at 37 °C and 160 rpm. Subsequently, cultures were adjusted to McFarland 0.5 in 2 mL of NaCl. To obtain final inoculum suspensions with approximately 5 × 10^5^ CFU/mL, the adjusted cultures were diluted in either CAMHB for HY-133 or CAMHB with 2% NaCl for oxacillin and supplemented with the appropriate amount of antimicrobial substance. MICs of the strains used for time-kill studies differed slightly depending on the growth phase tested (see Table S1 in the Supplementary Material). For a direct comparison of the activity of both antimicrobials against the WTs versus the corresponding SCVs, detailed time-kill kinetics were determined for 0.25, 0.5, 1, and 4 mg/L of HY-133 and oxacillin. Viability counts were carried out at 0, 1, 2, 4, 6, 8, 24, and 48 h of incubation at 37 °C and 160 rpm via plating culture aliquot dilutions on TSA in triplicate and incubation over night at 37 °C. A growth control without antimicrobial substance and a sterile control were included in each experiment. Mean colony counts (log_10_ of the numbers of CFU/mL) versus the time were plotted in graphs for each analyzed strain and antimicrobial (mean ± standard deviation).

### 4.5. Statistical Analysis

Statistical analysis and graphs were made using GraphPad PRISM software version 5.0 (GraphPad Software, LLC, San Diego, CA, USA). Changes in the number of CFU/mL after 1 h of incubation analyzed by time-kill curves were compared by one-way analysis of variance (ANOVA) with Dunnett’s test. A *p* value of ≤ 0.001 was considered significant.

## Figures and Tables

**Table 1:** Antimicrobial activities of HY-133 and oxacillin for 12 clinical wild type (WT) isolates compared with their clonally identical small-colony variants (SCVs).

Antimicrobial Agent	Growth Phase	Phenotype (No. of Strains)	Median MIC (mg/L) ^1^			Median MBC (mg/L) ^1^		
			50%	90%	Range	50%	90%	Range
HY-133	Stationary growth ^2^	WT (12)	0.12	0.5	0.12–0.5	0.12	0.5	0.12–0.5
		SCV (12)	0.25	0.5	0.12–0.5	0.25	0.5	0.12–0.5
	Logarithmic growth ^3^	WT (12)	0.25	0.5	0.25–0.5	0.25	0.5	0.25–0.5
		SCV (12)	0.12	0.5	0.12–0.5	0.12	0.5	0.12–0.5
Oxacillin	Stationary growth ^2^	WT (12)	0.5	1	0.25–2	0.5	1	0.25–2
		SCV (12)	0.25	1	0.25–1	0.25	1	0.25–2
	Logarithmic growth ^3^	WT (12)	0.5	1	0.25–1	0.5	1	0.25–2
		SCV (12)	0.25	0.5	0.12–1	0.25	1	0.25–1

^1^ 50% and 90%, MIC and MBC for 50% and 90% of strains tested, respectively. ^2^ Determination of MIC and MBC with direct colony suspension method. ^3^ Determination of MIC and MBC from log phase after 3 h of incubation. MIC and MBC were performed in triplicate for each strain and growth phase, and the determined medians were used to calculate the given MIC_50/90_ and MBC_50/90_ values and ranges.

**Table 2:** Comparison of antimicrobial activities of HY-133 and oxacillin against WT strain 6850, its gentamicin-selected SCV JB1, and its *hemB* mutant SCV IIb13.

Antimicrobial Agent	Growth Phase	Phenotype	Median MIC (mg/L)	Median MBC (mg/L)
HY-133	Stationary growth ^1^	6850 (WT)	0.12	0.12
		JB1 (selected SCV)	0.25	0.25
		IIb13 (mutant SCV)	0.12	0.12
	Logarithmic growth ^2^	6850 (WT)	0.25	0.25
		JB1 (selected SCV)	1	1
		IIb13 (mutant SCV)	0.25	0.25
Oxacillin	Stationary growth ^1^	6850 (WT)	0.5	0.5
		JB1 (selected SCV)	0.06	0.06
		IIb13 (mutant SCV)	0.06	0.06
	Logarithmic growth ^2^	6850 (WT)	0.5	0.5
		JB1 (selected SCV)	0.03	0.03
		IIb13 (mutant SCV)	0.03	0.06

^1^ Determination of MIC and MBC with direct colony suspension method. ^2^ Determination of MIC and MBC from log phase after 3 h of incubation. MIC and MBC of each strain and growth condition were determined in triplicate, and the calculated median MIC and MBC values are given.

**Table 3:** Times to achieve 50%, 90%, and 99.9% reductions in growth from starting inoculum when HY-133 or oxacillin was used.

Strain (Phenotype)	Growth Reduction	Time (h) when Respective Growth Reduction Was Reached for the Following Concentrations (mg/L) of Antimicrobial Used							
		HY-133				Oxacillin			
		0.25	0.5	1	4	0.25	0.5	1	4
OM299-1 (WT)	90%	NR	NR	1	1	8	4	2	2
	99%	NR	NR	1	1	NR	4	4	4
	99.9%	NR	NR	NR	1	NR	8	8	8
OM299-2 (SCV)	90%	NR	1	1	1	6	6	4	4
	99%	NR	NR	1	1	24	8	8	8
	99.9%	NR	NR	1	1	24	24	24	24
4652I (WT)	90%	NR	1	1	1	NR	4	4	2
	99%	NR	NR	1	1	NR	8	4	4
	99.9%	NR	NR	1	1	NR	24	8	8
4652II (SCV)	90%	1	1	1	1	4	4	4	2
	99%	1	1	1	1	8	4	4	4
	99.9%	NR	NR	1	1	NR	6	6	4
6850 (WT)	90%	NR	1	1	1	NP	NP	NP	NP
	99%	NR	1	1	1	NP	NP	NP	NP
	99.9%	NR	NR	1	1	NP	NP	NP	NP
JB1 (SCV)	90%	1	1	1	1	NP	NP	NP	NP
	99%	NR	NR	1	1	NP	NP	NP	NP
	99.9%	NR	NR	NR	1	NP	NP	NP	NP
IIb13 (SCV)	90%	1	1	1	1	NP	NP	NP	NP
	99%	NR	1	1	1	NP	NP	NP	NP
	99.9%	NR	NR	NR	1	NP	NP	NP	NP

NR, not reached; NP, not performed. Data were extracted from time-kill curve measurements.

**Table 4:** Characteristics of methicillin-susceptible *S. aureus* (MSSA) strains analyzed in this study.

Strain No.	Phenotype	Underlying Disease/Description	Source	Reference
A22616/5	WT	Osteomyelitis	Tissue ^1^	[39]
A22616/3	SCV		Tissue ^1^	[39]
OM1a	WT	Sternoclavicular joint arthritis with abscess	Tissue ^1^	[39]
OM1b	SCV		Tissue ^1^	[39]
OM184/1	WT	Acute osteomyelitis	Bone (distal radius)	[89]
OM184/2	SCV		Bone (distal radius)	[89]
OM299-1 ^2^	WT	Femur osteomyelitis	Tissue (femur)	[39]
OM299-2 ^2^	SCV		Tissue (femur)	[39]
OM420/1	WT	Knee arthrodesis-associated chronic osteomyelitis	Tissue (tibia)	[89]
OM420/3	SCV		Tissue (tibia)	[89]
4652I ^2^	WT	Acute osteomyelitis with tibia abscess	Abscess (tibia)	[89]
4652II ^2^	SCV		Abscess (tibia)	[89]
K3515I	WT	Sepsis	Blood	This study
K3515II	SCV		Blood	This study
A9380II	WT	Lumbar spondylitis	Swab (lumbar disc)	This study
A9379I	SCV		Swab (lumbar disc)	[89]
OM372/1	WT	Chronic osteomyelitis	Tissue (femur)	This study
OM372/2	SCV		Tissue (femur)	[89]
14799	WT	Chronic osteomyelitis	Tissue (femur exostosis)	This study
OM40/1	SCV		Tissue (femur exostosis)	[89]
OM234	WT	Hip osteoarthritis	Swab (joint)	This study
OM235/2	SCV		Swab (bone)	This study
A5382I	WT	Hip TEP infection	Swab (joint)	This study
A5382III	SCV		Swab (joint)	This study
6850 ^2^	WT	Skin abscess	-	[94]
JB1 ^2^	SCV	SCV, in vitro selected with gentamicin from 6850	-	[88]
IIb13 ^2^	SCV	Δ*hemB* (*hemB::ermB*) mutant from 6850	-	[95]
ATCC 29213	WT	Reference strain, *S. aureus* subsp. *aureus*	Wound	ATCC

^1^ Not further classified. ^2^ Strains used in time-kill studies. ATCC, American Type Culture Collection (LGC Standards GmbH, Wesel, Germany); QC, quality control; TEP, total endoprosthesis.

## Supplementary Materials

The following is available online at http://www.mdpi.com/1422-0067/20/3/716/s1, Table S1: MIC and MBC values of HY-133 and oxacillin for clinical *S. aureus* pairs OM299 and 4652 comprising WT isolates and their clonally identical SCVs as well as of the 6850-derived triplet for the evaluation of time-kill curves.

## Abbreviations

CBD	Cell wall-binding domain
CHAP	Histidine-dependent amidohydrolases/peptidases
EAD	Enzymatically active domain
MBC_50/90_	Minimum bactericidal concentration required to kill 50% and 90%, respectively, of the tested organisms
MIC_50/90_	Minimum inhibitory concentration required to inhibit growth of 50% and 90%, respectively, of the tested organisms
SCV	Small-colony variant
TMP-SXT	Trimethoprim-sulfamethoxazole
WTA	Wall teichoic acid
WT	Wild type
