# Supplementary material for: In Vitro Activity of the Bacteriophage Endolysin HY-133 against Staphylococcus aureus Small-Colony Variants and Their Corresponding Wild Types
Source: Int J Mol Sci. 2019 Feb 7;20(3):716. doi: 10.3390/ijms20030716 (PMC6387228; doi:10.3390/ijms20030716)
Supplement: Supplementary file 1 [file ijms-20-00716-s001.pdf]

International Journal of *Molecular Sciences*

MDPI

In vitro Activity of the Bacteriophage Endolysin HY-133 against *Staphylococcus aureus* Small-Colony Variants and their Corresponding Wild Types

Schleimer et al.

Supplementary Material

Table S1. MIC and MBC values of HY-133 and oxacillin for clinical *S. aureus* pairs OM299 and 4652 each comprising WT isolates and their clonally identical SCVs as well as of the 6850-derived triplet for the evaluation of time-kill curves.

Antimicrobial agent	Strain pair	Strain (phenotype)	MIC and MBC [1] in mg/L (stationary growth [2])	MIC and MBC [1] in mg/L (logarithmic growth [3])
HY-133	OM299	OM299-1 (WT)	0.12	0.5
		OM299-2 (SCV)	0.25	0.12
	4652	4652I (WT)	0.12	0.25
		4652II (SCV)	0.25	0.12
	6850	6850 (WT)	0.12	0.25
		JB1 (SCV)	0.25	1
		IIb13 (SCV)	0.12	0.25
Oxacillin	OM299	OM299-1 (WT)	0.25	0.25
		OM299-2 (SCV)	0.25	0.25
	4652	4652I (WT)	0.5	0.5
		4652II (SCV)	0.25	0.25

[1] MIC and MBC values were identical for these strains.

[2] Determination of MIC and MBC with direct colony suspension method.

[3] Determination of MIC and MBC from log phase after 3 h of incubation.

MIC and MBC of each strain and growth condition were determined in triplicate and the median MIC and MBC values were calculated.
